# Progress in Medicine

**Published:** 1899-11-11

**Authors:** 


### PROGRESS IN MEDICINE.

### DISEASES OF THE DIGESTIVE ORGANS.

(*Continued from page 80.*)

**Ulcer of the Stomach.**—When cancer of the stomach has been diagnosed the result not infrequently shows the disease to have been in some other organ, the stomach itself being really free. Lenetz[8] explains this by the absence of any single symptom, or symptoms which are indicative of cancer of the stomach alone. The presence of large immotile bacteria is, however, strong evidence, especially if a tumour can be made out, but the absence of H.Cl. and the occurrence of coffee-ground vomit or of lactic acid may be due to other causes. The occasional discovery of syphilis of the stomach in cases regarded as malignant should not be forgotten. R. L. Wood[9] has reported such an instance where pain and hæmatemesis with stercoraceous vomiting was thought to be due to malignant ulceration, but the symptoms and subsequent relapses cleared up under iodide of potash. J. W. Dalgliesh[10] notices three similar cases of obscure stomach affections which disappeared under anti-syphilitic remedies. In two of these there was a tumour, and in one severe hæmorrhage.

**Hyperchlorhydria.**—Esdall[11] gives a summary of the views which have been held on the continuous presence of gastric juice with H.Cl. in the stomach. Schreiber and his followers think this is normal, while Riegel and others look upon it as an evidence of disease. However, only 6·7 per cent. of the reported cases where no disease could be detected have more than a very small amount of acid, and many of these may have been due to the irritation of the tube. When larger amounts occur he thinks that a severe type of hyperchlorhydria exists. It does not appear that the condition is always due to motor weakness from either pyloric or sub-pyloric stenosis, though Robin has taken that view, and others have referred it to spasm of the pylorus. Another theory is that the excess of acid causes the motor weakness. Esdall concludes that continuous secretion may exist as a neurosis without organic disease, or may result from changes in the mucous membrane produced by ulcer, cancer, or obstruction.

Simple hyperchlorhydria may produce no local symptoms according to Boardman Reed.[12] There may be constipation or diarrhœa and flatulence, and nervous symptoms such as inability to sleep after four or five a.m. There is sometimes burning pain during digestion, and spasm of the pylorus and dilatation may result in bad cases. The stomach contents after a test breakfast show much mucus and an excess of H.Cl., such as ·1 per cent. or more, but smaller quantities, if persistent and accompanied with much mucus, are also indicative of the affection. Pain is often relieved by taking bland forms of albuminous food or by alkalies. Lemoine[13] recognises three types, the paroxysmal, the ordinary, and the continuous forms. In the first a violent burning pain is suddenly felt in the pit of the stomach, and lasts for half an hour or more. Morphia and a large dose of bicarbonate of soda may cut short these occasional attacks. In the second type pain is felt two or three times a day and during the night. For this he also employs large doses of soda half an hour before the time when the pain commences, but, as this treatment if long continued would aggravate the disease, he soon replaces the soda by magnesia and salicylate of bismuth, and if fermentation is present recommends lavage. Wegele[14] employs an emulsion of bismuth of 5 per cent. or a 1 per cent. solution of nitrate of silver. This is swallowed, and massage employed over the stomach for a few minutes, when the solution is drawn off by a stomach tube and the viscus washed out. He claims satisfactory results from this remarkable procedure, which he varies for the different types of chronic gastric affections. In dilatation J. A. Lichty[15] employs at first a diet of milk and raw eggs, keeping his patients on their backs after each meal to avoid any downward pressure. He gradually substitutes solid food, and endeavours to fatten his patients, but avoids potatoes, white bread, much fat, and raw fruits. Massage, faradism, and nux vomica are also valuable aids. Acute gastric catarrh, such as arises from overeating, demands the utmost care in diagnosis; afterwards, as Boardman Reed[16] remarks, the chief difficulty is to get patients to abstain from food. He finds most benefit is gained by a cold or hot compress over the stomach, an emetic of plain warm water, and afterwards single tablespoonfuls of merely cool water by the mouth. A sixth of a grain of calomel taken dry on the tongue may be given, and repeated every half-hour in bad cases till it produces copious yellow stools, even when watery offensive motions have been passed from the beginning. When the acute stage is over bismuth may be useful. Alcoholic cases need feeding as soon as possible, but much liquid by the mouth to quench the thirst is most harmful. In a paper on quantitative tests he[17] shows the great practical distinction between chronic dyspepsias with excess of H.Cl. and those with a deficiency. For the latter will benefit by pepsin and H.Cl. as medicines, with a stimulating diet and massage of the stomach; while the former require full doses of alkalies, and bland soothing foods. He advises Toepher's test method to measure (*a*) the free H.Cl., (*b*) the total of all acids present, and (*c*) the combined H.Cl. Thus (*a*) 10 cc. of the stomach contents are taken, and two or three drops of a half per cent. solution of dimethyl-amido-benzol are added, which gives a brilliant carmine with free H.Cl. A decinormal solution of caustic soda is added till the colour begins to fade. If 2·5, 4·, or 5· cc. are required, the amount of free H.Cl. present is said to be 25, 40, or 50 per 100 cc. The actual percentage of H.Cl. can be expressed by multiplying by ·00365, which is the amount each cc. of the soda solution neutralises. Again (*b*), if two or three drops of 1 per cent. alcoholic solution of phenolthalein are added to 10 cc. of the contents, and the soda solution again dropped in till a red colour appears, and no darkening occurs on further addition, we get a measure of the total acids present. If 8 or 9 cc. is used, we say the total acidity is 80 or 90. Finally (*c*), he adds to a third portion (10 cc.) two or three drops of a 1 per cent. watery solution of alizarin and neutralises as before to a clear violet tint. This gives the amount of all acid present, less the combined H.Cl. Thus, if this result is 60 or 70, by subtracting it from that of the phenolthalein test, we get the amount of combined H.Cl. as 20. It is thus easy to obtain figures representing the free H.Cl., the total acidity, and the combined H.Cl.

**Liver.**—Fringuet[18] records an outbreak of infective jaundice in a school; seven children out of thirty were affected, but all recovered quickly, and no cause could be discovered for the outbreak. There was general malaise with vomiting, the liver was enlarged and tender, and the spleen swollen. A. S. Abbott[19] summarises the known facts as to the relation of the bile to infectious and intestinal fermentations as follows: Outside the body it has, as a whole, no germicidal and but doubtful antiseptic qualities. In a few forms of infection the micro-organisms persist in the bile actually longer than elsewhere. In the case of serpent poison the bile neutralises the poison, and protects to some extent against subsequent infection. In rinderpest the bile of infected animals protects those into whom it is injected against attacks of the disease, but it is suggested that this is due to the action of the infected bile on the tissues into which it is injected. The organisms grow at this spot, and are probably prevented from spreading by the irritated tissues around, though their toxins are slowly absorbed. Thus, the known actions of the bile on infections are very few. Arnold, indeed, reports that frogs survive injections of strychnia when mixed with bile, which would otherwise be fatal, and suggests its action may explain the stronger effect of alkaloids given subcutaneously instead of by the mouth.

Instances of infection of the gall bladder in typhoid fever have been often reported of late. Ryska[20] gives the history of three patients in whom distension took place in the course of the fever, with more or less tenderness, and, in two cases, enlargement of the liver. All three made a good recovery. Camac, in a similar case, aspirated the gall bladder, and then performed cholecystotomy with a fatal result. There was no calculus present, but bacilli were present in the fluid contents of the gall bladder.

Appendicitis is rarely complicated with pyæmic abscesses in the liver, but an instance is given by W. F. Haslem[21] as occurring in a boy of eight years. The liver was enlarged and full of small abscesses containing fœtid pus, while the appendix was gangrenous and perforated. There had been abdominal symptoms with rigors, pain, and tenderness over the liver, which projected some three inches beyond the costal margin.

[8] Internat. Med. Mag., Aug. [9] Lancet, Sept. 23. [10] Lancet, Aug. 12. [11] Amer. Jour. Med. Sci., June. [12] Internat. Med. Mag., Aug. [13] Treatment, June 8. [14] La Semaine Med., April 26. [15] Internat. Med. Mag., April. [16] Ditto. [17] Internat. Med. Mag., May. [18] Brit. Med. Jour., Aug. 26. [19] Columbus Med. Jour., May 2. [20] Med. Chron., July. [21] Birmingham Med. Rec., Sept.

### NEUROLOGY.

**Transverse Section of the Spinal Cord.**—Schäfer[1] has recently carried out a series of experimental sections of the spinal cord, mostly in the monkey. The physiological results of hemisection of the cord were as follows: Complete motor paralysis of all parts supplied with nerves below the lesion on the same side. The limbs on the paralysed side were swollen and warm (vasomotor paralysis and lessened outflow of lymph) and the skin was dry. The knee jerk was exaggerated. Sensation was dulled, but not lost, on the same side as the lesion. After a few days this defect of sensation passed off. There was no sign of paralysis, either motor or sensory, on the side opposite to the hemisection. Purely voluntary movements are not recovered, though all the ordinary associated movements of the limbs are restored. Excitation of the motor cortex of the opposite cerebral hemisphere produces, as a rule, no movements of the limbs which have been paralysed, even though the associated movements have long returned. Lesions of the cerebellar tracts produced nodrious symptoms. After hemisection the cells of Marke's column below the lesion undergo chromatolysis, and eventually almost complete atrophy. This atrophy is equally complete if the lesion is confined to the dorsal and ventral cerebellar tracts. It is, therefore, to be inferred that Clarke's column gives origin not only to the dorsal (direct), but also to the ventral (Gowers') cerebellar tracts. Clarke's column has also an important relationship to the fibres of the pyramidal tract, for after lesions involving these fibres degenerative fibres are seen passing from the pyramidal tract below the lesion towards Clarke's column. No degenerated fibres are traceable from the pyramidal tract into the anterior horn, or into any part of the grey matter other than the base of the posterior horn or Clarke's column.

**Cerebellum.**—Risien Russell[2] has given a useful summary of a portion of the work he has done in the cerebellum. Removal of one-half of the cerebellum induces a state of increased excitability of the cortex of the opposite cerebral hemisphere. This increased excitability is manifested by the mode of behaviour of the muscles when convulsions are produced by the intravenous injection of absinthe, and also by difference in the movements produced by excitation of the cerebral cortex with the faradic current. It appears, then, that normally one-half of the cerebellum exerts an inhibiting or controlling influence on the neurons of the cortex of the opposite cerebral hemisphere, and that with ablation of one-half of the cerebellum this influence is removed, and increased excitability of the opposite cortex results. Such influences probably reach the cortex of the opposite cerebral hemisphere by means of the superior cerebellar peduncles; for, after ablation of one-half of the cerebellum, the fibres of the superior cerebellar peduncle on the same side degenerate, and these degenerated fibres are found to decussate and to terminate in the region of the red nucleus, and in the optic thalamus.

The head is inclined to the side of the lesion, so that the ear and shoulder are approximated to each other. In man the head may furthermore be rotated on its vertical axis, so that the chin points to the healthy side; that is, away from the cerebellar lesion. Rotation of the subject about its longitudinal axis is sometimes observed after removal of half of the cerebellum, but is very rarely met with as a result of cerebellar disease in man. The manner of rotation may be explained by comparison with the rotation of a screw, which enters the head of the subject; then with a right-sided lesion the subject rotates like a screw entering an object, while with a left-sided lesion the mode of rotation is like a screw coming out of an object.

General titubation and reeling are constant symptoms after removal of half of the cerebellum in animals, and are common in disease of the cerebellum in man. In man the direction of reeling is very unreliable as an indication of the side of the cerebellum diseased. After experimental lesions a turning of the eyes away from the side of the lesion is noticed, but this displacement is rare in cerebellar disease in man. In man abnormal positions of the eyes may be due to pressure on the nerves supplying the ocular muscles, or to secondary infiltration of the pores by a growth originating in the cerebellum. The sixth nerve is most liable to suffer owing to its slender size and long course. After ablation of one-half of the cerebellum in animals there is a true motor paresis of the limbs on the side of the lesion, but this symptom is rarely met with in cases of cerebellar tumour in man, probably owing to compensation going on hand in hand with the slowly-produced defects of disease. It is, however, much more common in cases of cerebellar abscess, where the process is more acute than in tumour. After ablation of half of the cerebellum both knee-jerks are increased, though that on the side of the lesion is the more exaggerated. In man a similar condition is sometimes met with, in other cases there is no difference between the two sides, whilst in some cases both knee-jerks are abolished. Clinically there are two symptoms which, if present, indicate the side of the cerebellum affected by the tumour. These are facial paralysis of the peripheral type and deafness; both occur on the same side as the tumour, and they also indicate that it is situated on the anterior part of the posterior fossa of the skull.

**Plantar Reflex.**—It was pointed out by Babinski[3] that in lesions of the pyramidal tracts of the spinal cord the normal plantar reflex, namely, flexion of the great toe followed by flexion of the other toes, was replaced by an extension of the great toe followed by that of the others; the flexor type was replaced by an extensor one. Collier[4] has given the results of a large number of observations undertaken to test these statements. He found that in infants the plantar reflex is naturally of the extensor type and continues as such until the child is able to walk, but in feeble, and especially in rachitic children it may be delayed until the fourth year. After this the reflex is nominally of the flexor type. In cases of total transverse lesion of the spinal cord the plantar reflex may be entirely absent, but if present it takes the form of sluggish extension. In 36 cases of myelitis 34 showed the extensor response on both sides. In 36 cases of organic hemiplegia, 28 showed an extensor response in the paralysed limb, whilst the sound side gave the normal flexor response, and 3 cases gave the extensor type on both sides. In 16 cases of cerebral tumour not involving the pyramidal system the responses were of the normal flexor type. In 35 cases of functional nervous disease the flexor response was obtained in 28, while in the remaining 7 it was abolished. In 28 cases of tabes 5 gave rise to irregular and uncertain movements, 11 showed an absence of the plantar reflex, while 9 gave the normal type of flexor response. In 15 cases of peripheral neuritis 6 exhibited no reflex, while 9 gave a slight response of the flexor type. In chorea and neurasthenia the normal flexor plantar reflex was obtained. The importance of these reflexes in the diagnosis of nervous affections is very considerable, and this new knowledge, which we owe to Babinski and Collier, will greatly facilitate the differential diagnosis of, for instance, disseminated sclerosis and hysteria. The general result is as follows: An extensor type, *i.e.*, extension of the great toe at the metatarsal phalangeal joint, of plantar reflex indicates a lesion in the pyramidal system (*i.e.*, motor cerebral cortex or pyramidal tract) unless the patient is an infant. On the other hand, an absence of the reflex indicates either a lesion in the reflex arc (*e.g.*, in tabes, peripheral neuritis) or is due to hysteria. To this statement there is an exception—in some cases of total transverse lesion of the spinal cord the plantar reflex is absent.

[1] Schäfer, Jour. of Phys., Mar., 1899. [2] Risien Russell, Dublin Jour. of Med. Sci., July, 1899. [3] Babinski, La Semaine Méd., Juillet 28, 1898. [4] Collier, Brain, Spring, 1889.

